# Opioid-Induced Immunomodulation: Consequences for the Experimental Coxsackievirus B3-Induced Myocarditis Model

**DOI:** 10.3390/biology9100335

**Published:** 2020-10-13

**Authors:** Kathleen Pappritz, Sophie Van Linthout

**Affiliations:** 1 Campus Virchow Klinikum (CVK), Berlin Institute of Health Center for Regenerative Therapies (BCRT), Charité—Universitätsmedizin Berlin, 13353 Berlin, Germany; sophie.van-linthout@charite.de; 2 German Center for Cardiovascular Research (DZHK), Partner Site Berlin, 10115 Berlin, Germany

**Keywords:** coxsackievirus B3 (CVB3)-induced myocarditis, opioids, immunomodulation

## Abstract

**Simple Summary:**

Myocarditis is an inflammatory disorder of the heart mainly caused by viruses. To investigate viral myocarditis, the Coxsackievirus B3 (CVB3)-induced myocarditis model is the experimental model used since more than sixty years. In the pathogeneses of viral myocarditis, the subtle balance between pro-and anti-inflammatory immune responses is of great importance for disease manifestation. Parallel to the infection of the heart, experimental CVB3-induced myocarditis results in an infection of the pancreas, causing a severe burden for the challenged animals. In frame of animal welfare, application of analgesics is mandatory. So far, positive as well as negative effects of opioids on the immune system have been described. However, the impact of opioid application on the pathogenesis of experimental CVB3-induced myocarditis has not been investigated yet. Since examinations on disease pathways and new treatment options rely on established models to generate reproducible data, applicability of opioids in experimental CVB3-induced myocarditis needs to be carefully evaluated. For this purpose, we summarized published studies for 13 different opioids and discussed their potential impact on the CVB3-induced myocarditis model.

**Abstract:**

Myocarditis is an inflammatory disorder of the heart predominantly caused by infectious agents. Since more than sixty years, the Coxsackievirus B3 (CVB3)-induced myocarditis mouse model is the experimental model used to investigate viral myocarditis. The pathogenesis of viral myocarditis is conceptually a multiphase process, initiated by the infection of cardiomyocytes, followed by activation of the immune system, and resulting in myocardial fibrosis and left ventricular dysfunction. In parallel to the direct infection of the heart, CVB3 replicates in lymphatic organs such as the pancreas. Due to infection of the pancreas, the model of experimental CVB3-induced myocarditis is estimated as a severe burden for the challenged animals. Application of analgesics in frame of the animal welfare act (European directive 2010/63/EU) is more and more becoming a matter of debate. For this purpose, we summarized published studies for 13 different opioids and discussed their potential impact on CVB3-induced myocarditis. In addition, with this summary we also want to provide guidance for researchers beyond the myocarditis field to estimate the impact of opioids on the immune system for their specific model. In the literature, both immunosuppressive as well as immune-activating effects of opioids have been described, but examinations in experimental CVB3-induced myocarditis have still not been reported so far. Based on the existing publications, administration of opioids in experimental CVB3-induced myocarditis might result in more severe disease progression, including higher mortality, or a less pronounced myocarditis model, failing to be used for the establishment of new treatment options. Taken together, the applicability of opioids in experimental CVB3-induced myocarditis and in inflammatory models in general needs to be carefully evaluated and further investigated.

## 1. Introduction

### 1.1. The Experimental Model of Coxsackievirus B3-Induced Myocarditis

Myocarditis is an inflammatory heart disease, primarily caused by infectious agents leading to heart failure [1]. The enterovirus, coxsackievirus B3 (CVB3), is considered to be the most studied human pathogen of viral myocarditis [2]. About 60 years ago, a mouse model of experimental CVB3-induced myocarditis was established by intraperitoneal (i.p.) infection of mice with CVB3 [3,4]. Given the resemblance of the myocardial injury in those mice with that occurring in humans, this model has become the standard model to study virus-induced myocarditis [5].

Following viral entry via the coxsackie-adenovirus receptor, apoptosis of cardiomyocytes and other cardiac resident cells is initiated [6,7]. This leads to direct damage of myocardial tissue. In addition, the innate immune response is induced to provoke a defense reaction against the virus and to clean up cell debris, involving the release of inflammatory mediators such as cytokines (tumor necrosis factor (TNF)-α, transforming growth factor (TGF)-β, anti-viral interferon (IFN)-β and IFN-γ, and interleukins (IL-1β, IL-2, IL-6…) and chemokines (monocyte chemoattractant protein (MCP)-1, MCP-3…) [8]. These mediators trigger a vicious circle characterized by further infiltration of immune cells such as monocytes/macrophages, dendritic cells (DCs), natural killer cells (NK), and neutrophils into the myocardium and release of additional pro- and anti-inflammatory cytokines/chemokines [9]. In addition, generation of reactive oxygen species (ROS) also occurs, which promotes the further death of cardiac cells [10]. In parallel, inflammatory processes trigger the accumulation of collagen and extracellular matrix in the heart, resulting in left ventricle (LV) stiffening and finally in a reduction of cardiac function [11,12]. Besides the well-described direct infection of the heart in the context of experimental CVB3-induced myocarditis, there is cumulative evidence reporting primarily infection of lymphatic organs [13], including the pancreas [14,15]. Mediated by the ongoing pancreatitis, the virus also migrates from the pancreas into the heart via the activated/infected immune cells [15,16]. The spleen is also a target organ of CVB3 [15], and splenic B cells, CD4+ T cells and Mac-1 macrophages/monocytes, target cells of CVB3. Taken together, experimental CVB3-induced myocarditis is a multi-factorial process, where the subtle balance between pro- and anti-inflammatory responses determines disease progression and therefore experimental manifestation [17,18].

### 1.2. Relevance of Opioid Administration in Coxsackievirus B3-Induced Myocarditis

Due to the induced pancreatitis and associated weight decrease [19], suggested to be the result of malabsorption and no appetite, experimental CVB3-induced myocarditis is estimated to be a severe burden for the challenged animals. Invoking the European Directive (Directive 2010/63/EU), use of analgesics for severely stressed animals is mandatory. But, application of analgesics especially in inflammatory disease models is still a matter of debate. Nonsteroidal anti-inflammatory drugs are known to cause, in addition to their impact on the subtle balance between pro- and anti-inflammatory responses, severe side effects like increased cardiovascular risk [20,21] or adverse gastrointestinal events [22], which both are contradictory for their use in experimental CVB3-induced myocarditis. 

Related to opioids, both immunosuppressive as well as immune-activating effects have been described [23]. The immunosuppressive effects might cause the development of an increased sensitivity towards pathogens [24,25] or worse disease outcome [26,27]. This is supported by excellent review articles describing an increased number of opportunistic infections after chronic application of opioids [28,29], implying that opioid administration in experimental CVB3-induced myocarditis might lead to worsened disease outcomes accompanied by severe suffering of mice. Increased expression of anti-inflammatory mediators, like IL-4 [30,31] and IL-10 [32,33], reduced clinical score [34], and less inflammation [35] have further been reported following opioid administration. Moreover, the substance itself exerts therapeutic effects [36], which makes it impossible to adequately evaluate new treatment options.

In brief, the impact of opioids on the immune response is not fully explored and there are no data available for the use of opioids in CVB3-induced myocarditis mice. Therefore, this article aims to summarize the existing knowledge about different opioids commonly used in experimental research and to further discuss their potential effects in the context of experimental CVB3-induced myocarditis.

## 2. Results and Discussion

In the current article, the use and described immune-related effects of 13 different opioids in rodents is summarized in view of their potential use in experimental CVB3-induced myocarditis. In detail, 13 different opioids (morphine, buprenorphine, codeine, fentanyl, hydromorphone, methadone, nalorphine, naloxone, naltrexone, oxycodone, tapentadol, tramadol, and remifentanil), their dose, route of administration, duration, rodent species, and their impact on the immune system are stated in Table A1.

### 2.1. Classification of Opioids

**Figure 1:**
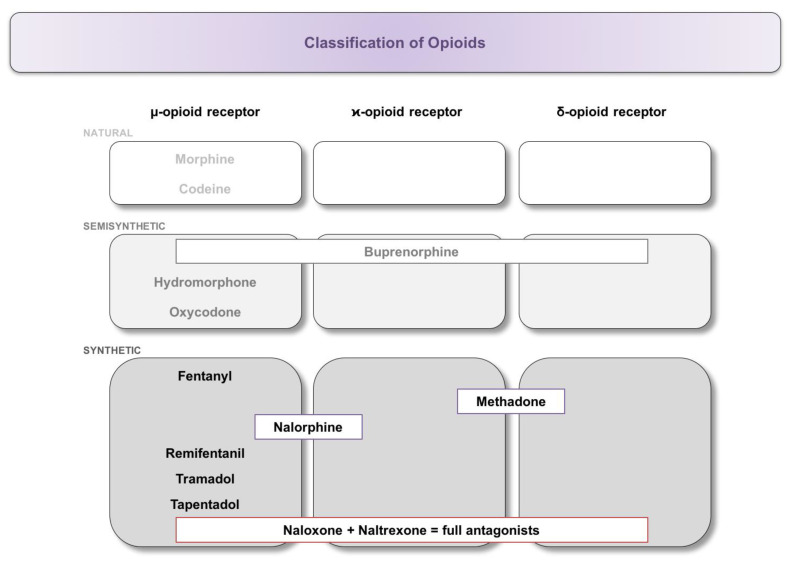
Classification of opioids and their binding affinity at the respective opioid receptor. Substances were classified into natural, semi-synthetic, and synthetic opiates, followed by further subdivision according to their binding to the μ-, κ-, or δ-opioid receptor.

Based on their chemical modification, substances are classified into natural opium alkaloids (morphine, codeine), semisynthetic opiates (buprenorphine, hydromorphone, oxycodone), and synthetic opiates (fentanyl, methadone, nalorphine, naloxone, naltrexone, tapentadol, tramadol, and remifentanil). Due to the different structures, differences in receptor binding (Figure 1) and subsequent effects on the immune system have been reported [23,37]. The exact mode of action is not the subject of this perspective article and has been described in detail elsewhere [38,39].

### 2.2. Impact of Opioids on the Immune System and the Heart of Healthy Animals

Besides the already known immunosuppressive effects of morphine, further insights into the complex impact of different opioids on immunity could be generated by our literature research. Effects of the administered substances ranged from immune suppression to immune activation, forcing a closer glance on existing publications. It is still a matter of debate, especially in preclinical research, how opioids influence disease progression in experimental models and the exact mode-of-action is still rarely known. Therefore, it is still challenging for researchers to conduct their studies in accordance to the guidelines of animal welfare, avoiding severe burdens for their animals on the one hand and providing robust data with established animal models on the other hand.

**Figure 2:**
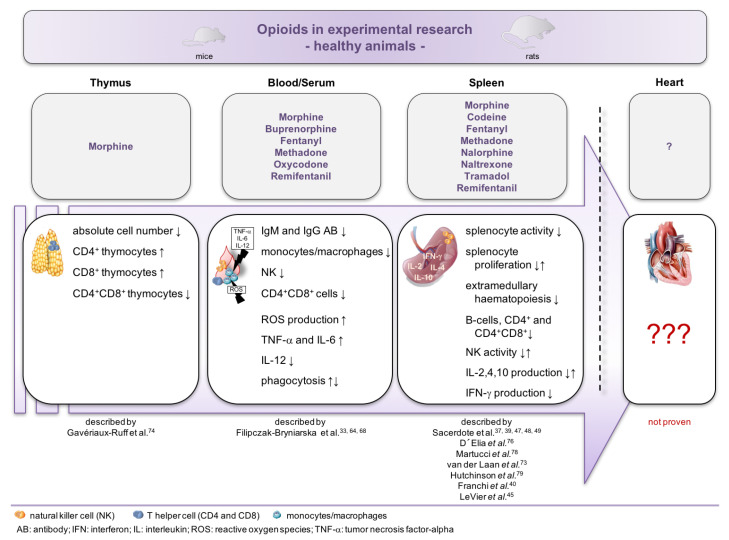
Impact of opioids on the immune system and the immune status of the heart in healthy animals. Overview of the reported effects of opioid administration in healthy mice. Despite extensive knowledge of the impact on peripheral mononuclear cells of the blood and spleen, nothing is known about the impact of opioids on the immune status of the heart.

One major concern of the analyzed studies is that the corresponding measurements have mostly been performed in healthy animals and not in disease models. It is undisputed that results under normal conditions are completely different compared to the respective disease model. Figure 2 summarizes the existing knowledge about the impact of different opioids on the immune system and the immune status of the heart in healthy rodents.

### 2.3. Impact of Opioids on the Immune System in Disease Models

**Figure 3:**
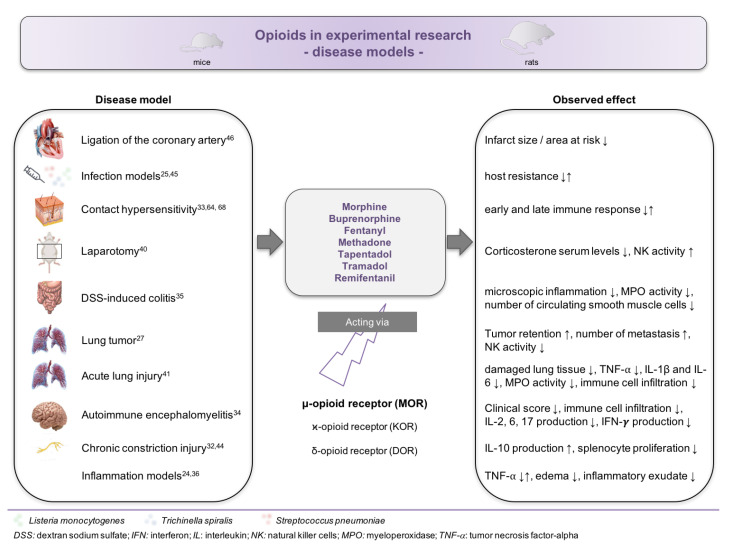
Impact of opioids on the immune system in disease models. Overview about the reported effects of opioid administration in various experimental disease models.

Little information exists related to the effect of opioid administration in disease models (Figure 3). In total, 16 studies have been conducted in disease models [24,25,26,27,32,33,34,35,36,40,41,42,43,44,45,46], including in models of lung cancer, contact hypersensitivity, infection, autoimmune encephalomyelitis, and surgery. In nine of those studies, rats were used [25,26,27,36,40,41,43,44,46], which does not allow a transfer of the results into murine models without critical evaluation. 

Apart from that, the published studies often did not apply uniform dosages or application routes, which hampers direct comparison of results and does not allow a prognosis for other models. Furthermore, Sacerdote et al. only investigated splenocyte proliferation, NK activity and IL-2 production to determine the immunosuppressive potential of the used opioids [37,47,48,49]. Especially in models of inflammatory disease, like CVB3-induced myocarditis, it is well established that the extent of the immune response is not mediated by only one cell population or cytokine but rather by an orchestra of innate and adaptive immunity components [10,11,50].

### 2.4. Impact of Opioids on the Heart

With respect to the heart, the impact of opioids on contractility, aging, ischemic pre-conditioning, and cardiogenesis involving several receptors has been reviewed [51]. In ischemic pre-conditioning rodent models, morphine exerted cardio-protective effects, as shown by reduced infarct size and area at risk [46]. Unfortunately, numerous studies have only been performed ex vivo in isolated hearts [52,53,54,55] or in vitro [56]. This complicates a direct extrapolation to the in vivo situation and particularly to an inflammatory disease as CVB3-induced myocarditis in which the immune system plays a predominant role.

The occurrence of opioid receptors in the heart and their cross-talk to the cardiac β-adrenergic receptors might explain the cardiac vascular side effects caused by chronic opioid abuse [57,58,59,60].

### 2.5. Impact of Opioids on Coxsackievirus B3-Induced Myocarditis

As described in the introduction, the pathogenesis of CVB3-induced myocarditis, of which all the established experimental models are conducted in mice [61,62], depends on the subtle balance between the pro- and anti-inflammatory response after infection. Interestingly, all listed opioids show either immunosuppressive or immune-activating effects, probably having an impact on the pathogenesis and severity of the CVB3 model itself. For example, treatment with fentanyl resulted in increased retention of lung tumor and tumor metastasis [27] or sensitization of the treated animals towards pathogens [24]. In contrast, tramadol induced therapeutic, anti-inflammatory effects in two models of inflammation, as indicated by lower number of edema and less inflammatory exudate [36].

With respect to cardiac function, none of the cited studies investigated the impact of opioids on cardiomyocytes, or fibroblasts. Indicative for the extent of LV contractility impairment and myocardial remodeling in viral myocarditis mice may be the generation of ROS, which leads to increased apoptosis of cardiomyocytes followed by reduced LV function [10]. Furthermore, ROS production and subsequent cardiomyocyte apoptosis leads to viral progeny release [63]. Oxycodone, morphine, buprenorphine, fentanyl as well as methadone all increase the production of ROS [33,64], which therefore represents a clear contraindication in experimental CVB3-induced myocarditis.

Additionally, previous own studies investigated the impact of various cell populations in the context of CVB3-induced heart failure [11,65], including monocytes/macrophages, NK cells, and DCs [50,66,67]. With respect to opioid administration, Filipczak-Bryniarska and colleagues intensively reported modulation of monocytes/macrophages and NK cells, and corresponding release of pro-inflammatory cytokines by LPS-stimulated macrophages [33,64,68]. Accordingly, an alteration of our established CVB3 myocarditis model after acute or chronic opioid application can´t be excluded.

## 3. Conclusions

Taken together, current evidence based on our literature search indicates that opioids have a modulating effect on the immune system. This implies that upon opioid administration the rodent model as such might be altered and the effect of the evaluated treatment option, influenced. Furthermore, knowledge about their impact on myocardial parameters is still insufficient.

Given the current state of the research, we assume that depending on the used substance, a more severe disease progression, including higher mortality and an overshooting inflammatory response, or a less pronounced myocarditis, which fails to provide robust data, are possible. Since animal studies have to be conducted in accordance with the animal welfare act, researchers are faced with a moral dilemma, revealing gaps in this field. Although there are publications on patients who reported heart failure symptoms after chronic opioid use, local animal welfare authorities require published evidence for the exclusion of analgesics in the experimental model of CVB3-induced myocarditis due to the severe burden. Investigations of disease pathways and new treatment options rely on established models to generate reproducible data, and even small changes on experimental protocols might result in less comparability of the examined effects. On the other hand, it is well known that the physiological and neuroendocrine effects of pain can impact research outcomes. This controversy between animal welfare and good scientific practice reveals a major challenge for the future, especially since animal-free alternative methods in viral myocarditis research are non-existent at the moment. Therefore, the applicability of opioids in experimental CVB3-induced myocarditis, and in inflammatory models in general, needs to be carefully evaluated and investigated further.

## 4. Significance Statement and Future Directions

Already some years ago, the discussion about the applicability of analgesics in CVB3-induced myocarditis started. Due to novel regulations in frame of the European Directive 2010/63/EU, the use of analgesics is required by the local animal welfare authorities and to receive the formal approval for experiments causing severe suffering like CVB3-induced myocarditis is impossible without application of analgesics. As described above, the experimental CVB3-induced myocarditis model was established 60 years ago via intraperitoneal application of CVB3 [3,4]. Besides direct infection of the heart, an infection of the pancreas also occurs, which causes the severity of the experimental CVB3 model [14,15]. Mediated by the ongoing pancreatitis, the challenged mice display reduced health status, and successful infection is associated with a strong decline in body weight [14]. A first attempt to reduce the severity of the CVB3 model was performed by Pinkert et al., who developed a new mouse model by attenuating CVB3 virulence in the pancreas [14]. Despite better health status and body weight, the novel H3N-375TS virus strain cannot be used for future translational research, because mice displayed no cardiac dysfunction. However, this is essential, enabling proper evaluation of the therapeutic impact of new treatment options. Therefore, other approaches, like application of certain opioids to achieve refinement of the CVB3-induced myocarditis model need to be investigated, especially since no target-specific strategies in viral myocarditis are established to date [1] and the clinical success of anti-inflammatory and immunomodulatory therapies is still limited [69]. Therefore, the established murine CVB3-induced myocarditis model needs to be improved to allow further valid research in this field.

## Appendix A

**Table A1:** Summary of used opioids in experimental research. In total, 2 natural opium alkaloids (morphine, codeine), 3 semisynthetic opiates (buprenorphine, hydromorphone, oxycodone), and 8 synthetic opiates (fentanyl, methadone, nalorphine, naloxone, naltrexone, tapentadol, tramadol, and remifentanil) were included. The table comprises their dose, route and duration of administration, rodent species, and opiate’s impact on the immune system under control or disease conditions.

Reference	Dosage, Route of Application, Duration	Species	Disease Model	End Points/Measured Parameters
*Morphine (prototype drug)*				
Bryant (1988) [70]	75 mg as a pellet; subcutaneously (s.c.) implanted → sacrifice 6, 24, 48, 72, 96, or 120 hours (h) later	mice	/	- spleen and thymus weight ↓ - adrenal gland weight ↑ - number of splenocytes at 48 and 72 h ↓ - Concanavalin-A (Con-A)-induced splenocyte proliferation at 48 and 72 h ↓ - lipopolysaccharide (LPS)-induced splenocyte proliferation at 24, 48, and 72 h ↓
Bryant (1988) [71]	75 mg as a pellet; s.c. implanted → sacrifice 6, 24, 48, 72, 96, or 120 h later	mice	/	- spleen and thymus weight ↓ between 24–72h - at 48 h: Con-A-induced splenocyte proliferation ↓ - at 120 h: Con-A-induced splenocyte proliferation ↑
Bryant (1991) [72]	75 mg as a pellet; s.c. implanted in adrenalectomized (ADX) mice → sacrifice 48h later	mice	/	sham ADX mice: - adrenal gland weight ↑ - spleen and thymus weight ↓ - LPS and Con-A-induced splenocyte proliferation ↓ ADX mice: - decrease spleen and thymus weight less pronounced - no reduction in splenocyte proliferation
van der Laan (1995) [73]	0.25–1.00 g/kg for 6 weeks (admixed into food)	rats	/	- liver, spleen, and pituitary gland weight ↓ - weight of lymph nodes ↑ - decrease in vacuolization of the hepatocytes ↓ (= glycogen storage ↓) - extramedullary hematopoiesis in the spleen ↓ - cell density in the medullary cords of the mesenterial lymph nodes ↑
Sacerdote (1997) [37]	2.5–20 mg/kg; once s.c.	mice	/	- splenocyte proliferation ↓ - NK activity ↓ - IL-2 production by splenocytes ↓
Schultz (1997) [46]	100 μg/kg; 3 times via intravenous infusion → 5 minutes (min) infusion time → sacrifice after 2h reperfusion period	rats	ligation of the left coronary artery (BUT: no sham control)	- infarct size/area at risk ↓ = therapeutic effect; but no sham animals included
Gavériaux-Ruff (1998) [74]	generation of μ-receptor knock-out (MOR) mice → daily application of 20–100 mg/kg via intraperitoneal injection (i.p.) for 6 days (d)	mice	/	WT animals: - atrophy of spleen and thymus - absolute cell number in the spleen ↓ - splenic NK activity ↓ - absolute cell number in the thymus ↓ - percentage of CD4^+^ und CD8^+^ thymocytes ↑ - percentage of CD4^+^CD8^+^ thymocytes ↓ MOR animals: - no modulatory effects observed
De Waal (1998) [25]	200 or 500 mg/kg food; administration up to 42d	rats	infection with *Listeria monocytogenes* or *Trichinella spiralis*	- serum corticosterone levels not affected - IgM and IgG antibody titers unchanged - clearance of *Listeria monocytogenes* bacteria in the spleen not affected- host resistance to *Trichinella spiralis* ↓
Filipczak-Bryniarska (2012) [33]	healthy animals: 20 mg/kg; i.p.; two times within 24 h for 5d disease model and transfer experiments: 20 mg/kg; i.p.; two times within 24 h for up to 7d → application in the induction and effector phase	mice	healthy mice + contact hypersensitivity model (CHS; pre-treatment with opioids)	healthy mice: - humoral immune response (=number of plaque forming cells) ↓ - serum levels of IgM and IgG antibodies ↓ - ROS production by macrophages ↑ - percentage of CD4^+^CD8^+^ cells in the blood ↓ - percentage of monocytes in the blood ↓ - expression of cell surface markers for antigen presentation on peritoneal macrophages ↓ - production of TNF-α and IL-6 by macrophages ↑ application in the induction phase of CHS: - early immune response after 2 h ↓ - late immune response after 24 h ↓ application in the effector phase of CHS: - early immune response after 2 h ↑ - late immune response after 24 h ↑
*Buprenorphine*				
Gomez-Flores (2000) [75]	0.66 nmol; single application in the mesencephalon periaqueductal gray	rats	/	- NK activity not changed - splenic TNF-α and NO expression not changed - phagocytosis not changed - plasma levels of adrenocorticotropic hormone and corticosterone ↓ BUT: - severe burden of the animals due to the operation - application route is not possible in experimental CVB3-induced myocarditis
D´Elia (2003) [76]	0.2 mg/kg*d^−1^ via an osmotic pump → application for up to 10d	mice	/	- corticosterone serum level at day 1 ↑ - corticosterone serum level at day 5 ↓ - ratio of CD4^+^ thymocytes at day 5 and 10 ↓ - ratio of CD8^+^ thymocytes at day 5 and 10 ↑ - splenocyte proliferation at day 5 ↑
Rätsep (2013) [77]	0.05 mg/kg, s.c., twice a day → observation until d7 after the surgery	mice	no real disease model; test of analgesics for telemeter implantation	- recovery of body weight at d6 post-surgery - food intake the first 3 days ↓ - daytime mean arterial pressure ↓ - night time mean arterial pressure ↓ - heart rate ↓
Franchi (2007) [40]	0.1 mg/kg, s.c., twice (at the end of the surgery and 5h later) → sacrifice 60 min after last the application	rats	laparotomy	- corticosterone serum level ↓ - NK activity ↑ - number of lung metastasis ↓ (=therapeutic effect)
Blennerhasset (2017) [35]	healthy animals: 0.05 mg/kg s.c. twice daily for 5d disease model: 0.05 mg/kg s.c. twice daily for 8d	mice	healthy mice + dextran sodium sulfate (DSS)-induced colitis	healthy mice: - no effect on food and water intake application in DSS-induced colitis: - microscopic inflammation ↓ - MPO activity ↓ - inhibition of smooth muscle cellular hyperplasia - number of circulating smooth muscle cells in the colon ↓ - axon area of the neuromuscular layer (colon) ↓ = anti-inflammatory effect
Filipczak-Bryniarska (2018) [64]	healthy animals: 2 mg/kg i.p.; one dose within 24 h for 7d disease model and transfer experiments: 2 mg/kg i.p.; one dose within 24 h for up to 11d → application in the induction and effector phase	mice	healthy mice + contact hypersensitivity model(CHS; pre-treatment with opioids)	healthy mice: - humoral immune response (=number of plaque forming cells) ↑ - ROS and NO generation by macrophages ↑ - intensity of fluorescence of cell surface markers for antigen presentation on macrophages ↓ - production of IL-12 by macrophages ↓ application in the induction phase of CHS: - early immune response after 2 h not changed - late immune response after 24 h ↓ application in the effector phase of CHS: - early immune response after 2 h ↓ - late immune response after 24 h not changed- late immune response after 48 h ↓
Filipczak-Bryniarska (2018) [68]	2 mg/kg i.p.; one dose within 24 h for 7d	mice	/	- phagocytosis of sheep red blood cells by macrophages ↑ - phagocytosis of zymosan-green by macrophages ↑
Jirkof (2019) [42]	0.009 mg/mL via drinking water for 3d	mice	osteotomy model	- no effect on food and water intake - model-specific pain behavior ↓ - no impact on bone fracture healing (bone volume fraction) and vessel formation
*Codeine*				
Sacerdote (1997) [37]	2.5–100 mg/kg; once s.c.	mice	/	- splenocyte proliferation not changed - NK activity ↓ - IL-2 production by splenocytes ↓
*Fentanyl*				
Martucci (2004) [78]	acute study: 0.25 mg/kg; once s.c. → sacrifice 60 min later chronic study: 7.5 μg/h (180 μg/day) via an osmotic pump → application up to 7d	mice	/	acute study: - lymphocyte proliferation ↓ - NK activity not changed - IL-2 und IFN-γ production by splenocytes not changed chronic study: - lymphocyte proliferation 24h and 3 days after the last application ↓ - NK activity 24h and 3 days after the last application ↓ - IL-2 production by splenocytes at day 3 after the last application ↓ - IFN-γ production by splenocytes at day 3 after the last application ↓
Shavit (2004) [27]	0.1–0.3 mg/kg; once s.c. → 6 h, 2 h, and 0 h before and 1 h after i.v. inoculation of tumor cells	rats	MADB106-induced lung tumor	- lung tumor retention after 3 weeks ↑ - number of metastases ↑ - NK activity ↓
Forget (2010) [26]	40 μg/kg; i.p.; 1h before surgery	rats	laparotomy +/− MADB106-induced lung tumor	laparotomy: - NK activity in non-operated and operated animals until day 8 ↓ laparotomy + MADB106-induced lung tumor: - number of metastases in non-operated animals ↑
Filipczak-Bryniarska (2012) [33]	healthy animals: 10 mg/kg; i.p.; two times within 24 h for 5 d disease model and transfer experiments: 10 mg/kg; i.p.; two times within 24 h for up to 7d → application in the induction and effector phase	mice	healthy mice + contact hypersensitivity model(CHS; pre-treatment with opioids)	healthy mice: - humoral immune response (=number of plaque forming cells) ↓ - serum levels of IgG antibodies ↓ - ROS production by macrophages ↑ - percentage of CD4^+^CD8^+^ cells in the blood ↓ - percentage of NK in the blood ↓ - expression of cell surface markers for antigen presentation on peritoneal macrophages ↓ - production of TNF-α and IL-6 by macrophages not changed application in the induction phase of CHS: - early immune response after 2 h not changed - late immune response after 24 h ↑ application in the effector phase of CHS: - early immune response after 2 h ↑ - late immune response after 24 h ↑
Molina-Martínez (2014) [24]	acute study: 0.001–0.1 mg/kg; once i.p → 10 min before LPS application → sacrifice 60 min later chronic study: 0.1 mg/kg every 8h i.p.; to complete 6 or 10 doses. → 10 min after the last opiate administration challenging to LPS → sacrifice 60 min later	mice	LPS-induced inflammation	acute study: - TNF-α release in the peritoneal cavity in response to LPS ↓ = anti-inflammatory effect mediated by intraperitoneal macrophages chronic study: - TNF-α release in the peritoneal cavity in response to LPS ↑ = pro-inflammatory effect = sensitization towards LPS application
*Hydromorphone*				
Sacerdote (1997) [37]	2.5–20 mg/kg; once s.c.	mice	/	- splenocyte proliferation not changed - NK activity not changed - IL-2 production by splenocytes not changed BUT: - splenocyte proliferation and IL-2 production had a tendency to be reduced after hydromorphone application → study showed no data for chronic application (administration several times)
*Methadone*				
van der Laan (1995) [73]	0.20–0.80 g/kg for 6 weeks (admixed into food)	rats	/	- weight testes and lymph nodes ↑ - decrease in vacuolization of the hepatocytes ↓ (= glycogen storage ↓) - extramedullary hematopoiesis in the spleen ↓ - cell density in the medullary cords of the mesenterial lymph nodes ↑ - serum levels of IgG antibodies ↑
LeVier (1995) [45]	healthy mice: 10–30 mg/kg; once s.c. → sacrifice 60 min later infection model: 10–30 mg/kg; once s.c. → bacterial challenge 60 min later → sacrifice at day 7 or 14	mice	healthy mice + infection with *Listeria monocytogenes* or *Streptococcus pneumoniae*	healthy mice: - splenocyte proliferation ↓ - cytotoxic T cell activity ↑ - change of the reticuloendothelial system - erythrocyte number dose-dependent ↑ - number of B-cells at 10 mg/kg ↓ - primary IgM of splenocytes at 20 mg/kg ↓ - number of CD4^+^ and CD4^+^CD8^+^ at 10 mg/kg ↓ infection model: - host resistance towards *Listeria monocytogenes* not altered - host resistance towards *Streptococcus pneumoniae* ↑
De Waal (1998) [25]	200 or 400 mg/kg food; administration up to 42d	rats	infection with *Listeria monocytogenes* or *Trichinella spiralis*	- serum corticosterone levels were not affected - IgG antibody titers at 400 mg/kg food ↑ - clearance of *Listeria monocytogenes* bacteria in the spleen not affected - host resistance to *Trichinella spiralis* not affected
Hutchinson (2004) [79]	1.5–3 mg/kg; once i.p. → sacrifice 120 min after application	mice	/	- splenocyte proliferation ↑
Filipczak-Bryniarska (2012) [33]	healthy animals: 30 mg/kg; i.p.; one dose within 24 h for 5d disease model and transfer experiments: 30 mg/kg; i.p.; one dose within 24 h for up to 7d → application in the induction and effector phase	mice	healthy mice + contact hypersensitivity model (CHS; pre-treatment with opioids)	healthy mice: - humoral immune response (=number of plaque forming cells) ↓ - serum levels of IgG antibodies ↓ - ROS production by macrophages ↑ - percentage of CD4^+^CD8^+^ cells in the blood ↓ - percentage of NK in the blood ↓ - percentage of monocytes/macrophages in the blood ↓ - expression of cell surface markers for antigen presentation on peritoneal macrophages ↓ - production of TNF-α and IL-6 by macrophages ↑ Application in the induction phase of CHS: - early immune response after 2 h ↑ - late immune response after 24 h ↑ Application in the effector phase of CHS: - early immune response after 2 h ↑ - late immune response after 24 h ↓
Kafami (2013) [34]	10 mg/kg*d^-1^ i.p. → start 3d after EAE induction → application for 33d (until day 35 = end point)	mice	MOG_35-55_-induced experimental autoimmune encephalomyelitis (EAE)	- severity of EAE ↓ - clinical score ↓ - infiltration of immune cells into bone marrow ↓ - production of IL-2 by T-cells ↓ - production of IL-6, IL-17 and IFN-γ by splenocytes ↓ = changed immune response and different pathogenesis of the model
*Nalorphine*				
Sacerdote (1997) [37]	2.5–20 mg/kg; once s.c.	mice	/	- splenocyte proliferation ↓ - NK activity ↓ - IL-2 production by splenocytes ↓
*Naloxone*				
Sacerdote (1997) [37]	2.5–20 mg/kg; once s.c.	mice	/	- splenocyte proliferation ↑ - NK activity not changed - IL-2 production by splenocytes ↑ BUT: - no analgesic effect = no use as pain killers
*Naltrexone*				
Sacerdote (1997) [37]	2.5–20 mg/kg; once s.c.	mice	/	- splenocyte proliferation ↑ - NK activity not changed - IL-2 production by splenocytes ↑ BUT:- no analgesic effect = no use as pain killers
*Oxycodone*				
Sacerdote (1997) [37]	2.5–20 mg/kg; once s.c.	mice	/	- splenocyte proliferation not changed - NK activity not changed - IL-2 production by splenocytes not changed BUT: - splenocyte proliferation and IL-2 production have a tendency to be reduced after oxycodone application → study showed no data for chronic application (administration several times)
Filipczak-Bryniarska (2018) [64]	healthy animals: 20 mg/kg i.p.; two doses within 24 h for 7d disease model and transfer experiments: 20 mg/kg i.p.; two doses within 24 h for up to 11d → application in the induction and effector phase	mice	healthy mice + contact hypersensitivity model (CHS; pre-treatment with opioids)	healthy mice: - humoral immune response (=number of plaque forming cells) not changed - ROS and NO generation by macrophages ↑ - intensity of fluorescence of cell surface markers for antigen presentation on macrophages ↓ - production of IL-12 by macrophages ↓ Application in the induction phase of CHS: - early immune response after 2h (↑) - late immune response after 24h not changed Application in the effector phase of CHS: - early immune response after 2 h not changed - late immune response after 24 h not changed - late immune response after 48 h ↓
Filipczak-Bryniarska (2018) [68]	20 mg/kg; i.p.; two doses within 24 h for 7d	mice	/	- phagocytosis of sheep red blood cells by macrophages ↑ - phagocytosis of zymosan-green by macrophages ↓
*Tapentadol*				
Franchi (2017) [32]	acute study: 20–30 mg/kg; once s.c. → sacrifice 120 min after application chronic study: 20 mg/kg; once s.c. for 4–7 days → sacrifice 24 h after application	mice	acute study: / chronic study: healthy animals + sciatic nerve chronic constriction injury (CCI; neuropathic model)	acute study (healthy animals): - production of IFN-γ, IL-2, IL-10 and IL-4 not changed chronic study—healthy animals: - production of IFN-γ, IL-2, IL-10 and IL-4 not changed after 4 + 7 days but:- production of IFN-γ after 4 days ↑ - production of IL-2 after 4 days ↓ chronic study—CCI model (compared to untreated CCI): - anti-hyperalgesic and anti-allodynic effect - IL-10 production of splenocytes ↑ = therapeutic effect - no reduction of IL-2, IL-4, and IL-10 production = altered disease model
*Tramadol*				
Sacerdote (1997) [48]	acute study: 0.1–80 mg/kg; once s.c. → sacrifice 60 min later chronic study: 20 mg/kg; 2 times daily; s.c. for 2 weeks → sacrifice 24 h after the last application	mice	/	acute study:- splenocyte proliferation ↑ - NK activity ↑ - IL-2 production by splenocytes ↑ chronic study: - splenocyte proliferation not changed - NK activity not changed - IL-2 production by splenocytes not changed BUT:- all parameters have a tendency to be reduced after tramadol application and were significantly altered after repeated application
Bianchi (1999) [36]	1.23–20 mg/kg; once i.p. → 15 min before induction of inflammation → sacrifice 3 h or 6 h after application	rats	yeast-induced inflammation + carrageenin-induced inflammation	yeast-induced inflammation (3 h after yeast application): - oedema ↓ - paw hyperalgesia ↑ carrageenin-induced inflammation (6h after sponge implantation): - volume of inflammatory exudate ↓ - PGE_2_ concentrations ↓- PGE_2_-like activity ↓
Sacerdote (1999) [47]	20 + 40 mg/kg; once i.p. → sacrifice 60min later	mice	/	- splenocyte proliferation ↑ - NK activity ↑
Tsai (2001) [44]	acute study: 20 + 40 mg/kg; once s.c. at d6 after surgery → sacrifice 60 min after application chronic study: 80 mg/kg; s.c. for 7d via osmotic pump → sacrifice at day 12 after hyperalgesia measurement	rats	acute study: CCI chronic study: CCI	acute study: - CCI rats exhibited dose-dependent reversal of paw withdrawal latency (thermal hyperalgesia) - NK activity not changed - splenocyte proliferation ↓ chronic study: acute study: - CCI rats exhibited dose-dependent reversal of paw withdrawal latency (thermal hyperalgesia) - NK activity not changed - splenocyte proliferation ↓
Gaspani (2002) [43]	20 + 40 mg/kg; s.c.; 3 doses → 30 min before surgery, 15 min after surgery, and 5 h after surgery	rats	laparotomy + MADB106-induced lung tumor	- at the dose of number of 40 mg/kg number of lung metastases ↓ - splenic NK activity of non-operated animals by 40 mg/kg ↑ - 20 + 40 mg/kg tramadol prevented reduced splenic NK activation after surgery
Rätsep (2013) [77]	20 mg/kg, s.c., once a day → observation until d7 after the surgery	mice	no real disease model; test of analgesics for telemeter implantation	- recovery of body weight at d5 post-surgery - food intake the first 3 days ↓ - daytime mean arterial pressure ↓ - night time mean arterial pressure ↓
Blennerhasset (2017) [35]	healthy animals: 20 mg/kg s.c. once daily for 5d disease model: 20 mg/kg s.c. once daily for 8d	mice	healthy mice + dextran sodium sulfate (DSS)-induced colitis and trinitrobenzene sulfonic acid (TNBS)-induced colitis	healthy mice: - no effect on food and water intake application in DSS-induced colitis: - microscopic inflammation not affected - MPO activity not affected application in TNBS-induced colitis: - progressive weight loss similar to TNBS alone - number of circulating smooth muscle cells in the colon unaffected - tramadol did not affected decrease in neuron number
Jirkof (2019) [42]	0.1 mg/mL (T_low_) and 1.0 mg/mL (T_high_) via drinking water for 3 days	mice	osteotomy model	- food and water intake at high concentration ↓ - model-specific pain behavior ↓, not further amelioration by T_high_ - higher limp score at 1h post-osteotomy - no impact on bone fracture healing (bone volume fraction) and vessel formation
*Remifentanil*				
Sacerdote (2001) [49]	50 μg/kg*min^-1^; infusion for 60 min → sacrifice 5 h after end of the infusion	rats	/	- lymphocyte proliferation in the blood↓ - NK activity in the blood ↓ - splenocyte proliferation ↓ - splenic NK activity ↓
Zhang (2014) [41]	0.04 mg/kg; for 10 min i.v. → sacrifice 8 h after challenging towards LPS	rats	LPS-induce acute lung injury (ALI)	- damaged lung tissue (thickening of the alveolar wall, interstitial oedema) ↓ - ALI-associated increase of pro-inflammatory cytokines (TNF-α, IL-1β, and IL-6) in the lung ↓ - reduced MPO activity - infiltration of neutrophils ↓ - immune cell infiltration in the lung ↓

α: alpha; *ADX*: adrenalectomized; *ALI*: acute lung injury; β: beta; *CCI*: chronic constriction injury; *CHS:* contact hypersensitivity model; *Con-A:* Concanavalin-A; CVB3: coxsackievirus B3; *d*: day; *DSS:* dextran sodium sulfate; *EAE*: experimental autoimmune encephalomyelitis; γ: gamma; *h*: hours; IFN-γ: interferon-γ *IL:* interleukin; *i.p.*: intraperitoneal injection; *LPS:* lipopolysaccharide; *MPO*: myeloperoxidase; *NK:* natural killer cell; *NO*: nitric oxide; µ: micro; *min:* minutes; *PGE_2_*: prostaglandin 2; *ROS:* reactive oxygen species; *s.c:* subcutaneously; TNF-α: tumor necrosis factor-α; *TNBS*: trinitrobenzene sulfonic acid; ↑: increase; ↓: decrease; →: followed by; +: positive; /: not studied.
